# MicroRNome Analysis Unravels the Molecular Basis of SARS Infection in Bronchoalveolar Stem Cells

**DOI:** 10.1371/journal.pone.0007837

**Published:** 2009-11-13

**Authors:** Bibekanand Mallick, Zhumur Ghosh, Jayprokas Chakrabarti

**Affiliations:** 1 Computational Biology Group, Indian Association for the Cultivation of Science, Calcutta, India; 2 Department of Medicine and Department of Radiology, Stanford University School of Medicine, Stanford, California, United States of America; Georgia Institute of Technology, United States of America

## Abstract

Severe acute respiratory syndrome (SARS), caused by the coronavirus SARS-CoV, is an acute infectious disease with significant mortality. A typical clinical feature associated with SARS is pulmonary fibrosis and associated lung failure. In the aftermath of the SARS epidemic, although significant progress towards understanding the underlying molecular mechanism of the infection has been made, a large gap still remains in our knowledge regarding how SARS-CoV interacts with the host cell at the onset of infection. The rapidly changing viral genome adds another variable to this equation. We have focused on a novel concept of microRNA (miRNA)–mediated host–virus interactions in bronchoalveolar stem cells (BASCs) at the onset of infection by correlating the “BASC–microRNome” with their targets within BASCs and viral genome. This work encompasses miRNA array data analysis, target prediction, and miRNA–mRNA enrichment analysis and develops a complex interaction map among disease-related factors, miRNAs, and BASCs in SARS pathway, which will provide some clues for diagnostic markers to view an overall interplay leading to disease progression. Our observation reveals the BASCs (Sca-1+ CD34+ CD45- Pecam-), a subset of Oct-4+ ACE2+ epithelial colony cells at the broncho-alveolar duct junction, to be the prime target cells of SARS-CoV infection. Upregulated BASC miRNAs-17*, -574-5p, and -214 are co-opted by SARS-CoV to suppress its own replication and evade immune elimination until successful transmission takes place. Viral Nucleocapsid and Spike protein targets seem to co-opt downregulated miR-223 and miR-98 respectively within BASCs to control the various stages of BASC differentiation, activation of inflammatory chemokines, and downregulation of ACE2. All these effectively accounts for a successful viral transmission and replication within BASCs causing continued deterioration of lung tissues and apparent loss of capacity for lung repair. Overall, this investigation reveals another mode of exploitation of cellular miRNA machinery by virus to their own advantage.

## Introduction

Severe Acute Respiratory Syndrome (SARS) is a new fulminant atypical pneumonia which emerged as a regional and global threat in 2002–2003 with a high mortality rate resulting from acute lung failure [1]. The disease causing agent has been identified as a novel coronavirus termed as SARS-associated coronavirus (SARS-CoV) [2], [3]. The SARS-CoV is an enveloped virus containing a single stranded, positive-sense RNA genome which encodes 14 putative open reading frames encoding 28 potential proteins [4], [5]. These include four structural proteins, spike (S) glycoprotein, matrix (M) protein, small envelope (E) protein, and nucleocapsid (N) protein [4]. These proteins have various roles in aiding the virus to enter the host and spread infection. While the incidence of new cases of SARS waned in 2003–2004, many aspects of SARS disease pathogenesis and host-pathogen interactions remain unsolved. Limited pathologic studies reveal that the major site of SARS-CoV infection and morbidity is the respiratory tract. The target organ of SARS is mainly lungs [6]. Among the various animal models that have been used to study the pathogenesis of SARS-CoV infection, the monkey model mimics the clinical course of SARS to a certain degree [7]. The cellular tropism of SARS-CoV in mouse lung has also been investigated by Ling *et al*., 2006 [8]. But still today, very little is known regarding the mode of SARS-CoV interaction with host cells at the onset of infection in the lungs at a molecular level, and also the cell types in which the primary viral infection and replication occurs.

The lung is an extremely complex, conditionally renewing organ which contains anatomically and functionally distinct epithelial stem cell populations which reside in distinct anatomical locations. The basal cells [9], Clara cells [10] and type-II pneumocytes [11] are the candidate stem/progenitor cells which can repair the injured lungs and contribute to local needs in times of tissue damage. Recently, Kim *et al*. have isolated a regional pulmonary stem cell population termed as bronchoalveolar stem cells (BASCs) residing at the bronchoalveolar duct junction of adult lungs [12]. These have been identified and characterized as CD34^+^ Sca-1^+^ CD45^-^ PE-CAM^−^ cells expressing both cytoplasmic Clara cell secretion protein (CCSP) and prosurfactant protein-C proteins, which are markers for Clara cells and type-II pneumocytes respectively [12]. We have searched for the evidence to establish BASC as the prime target cells of SARS infection initiation and replication.

To date, miRNA-mediated RNA interference is reported to be an essential tool to understand the regulatory pathways at molecular level that underlie infection biology [13], [14]. They not only participate in executive decisions but also perform much of the grunt work to micromanage protein output. Inspite of being such potential regulatory elements, miRNAs have so far missed attention in the pathogenesis of SARS-CoV infection.

MiRNAs are small (19–25 nucleotides) endogenous noncoding RNAs that have been shown to influence the abundance and translational efficiency of cognate mRNAs [15], [16]. Viruses, which typically employ many components of the host gene expression machinery, also encode miRNAs. On the contrary, miRNA-biogenesis pathway poses some serious problem for RNA viruses and a group of DNA viruses (poxviruses) to encode miRNAs [14]. SARS-CoV being an RNA virus is assumed not to encode its own miRNAs. But in due course of evolution, the virus might have developed certain highly sophisticated molecular mechanisms to exploit the cellular biosynthetic machinery of host cells and elude the cellular defense mechanisms. It is most likely that in certain situations SARS-CoV uses the cellular miRNA transcripts in order to foster their own agenda.

Given the proposed involvement of BASCs in SARS disease [8], elucidation of the miRNA-mediated regulatory mechanisms responsible for making BASCs the prime targets of SARS infection might provide new avenues to explore the underlying mechanisms of SARS infection.

### Onset of SARS infection

The entry of SARS-CoV into cells is mediated through interaction between spike (S) glycoprotein of the virus and angiotensin-converting enzyme 2 (ACE2), the primary receptor of SARS-CoV on the host cell [17]. ACE2 is a homolog of angiotensin-converting enzyme (ACE), which plays an important role in the renin–angiotensin system for blood pressure homeostasis. Recent studies of Li *et al*. [18] have shown a protective effect of ACE2 against experimental lung fibrosis through its ability to degrade local tissue angiotensin II (ANG II) in response to bleomycin. Furthermore, in the pathogenesis of lung fibrosis, they have proposed the involvement of the down regulation of ACE2 as an integral component of the sequence of events leading to lung collagen deposition. The molecular mechanisms responsible for the loss of ACE2 in lung fibrosis are currently under investigation. A typical clinical feature associated with SARS is also pulmonary fibrosis and associated lung failure whose underlying mechanism still remains elusive [19]. Investigations leading to the underlying molecular mechanisms responsible for the loss of ACE2 in pulmonary fibrosis might provide a clue to the reason behind fibrosis associated lung injury in SARS.

Several reports suggest that there is a close interaction between SARS-CoV and respiratory epithelia which play an important role in the genesis of SARS [20]. Jia *et al*. [21] have investigated interactions between SARS-CoV and human airway epithelia and indicated that the state of cell differentiation and ACE2 expression levels are important determinants of the susceptibility of human airway epithelia to infection. To date, the molecular switches regulating the state of cellular differentiation of an infected cell and ACE2 expression have not been studied properly. In this work, we have proposed the role of miRNA in modulating the expression of ACE2 along with the stage specific cellular differentiation at the site of infection initiation. Further we have tried to focus on the role of SARS-CoV which dictates these events for its successful transmission and replication.

The POU-homeodomain transcription factor Oct-4 (Pou5f1) plays a central role in self-renewal, pluripotency, and lineage commitment. Alterations in Oct-4 expression promote differentiation and leads to the specification of ectodermal, endodermal or mesodermal primitive progenitors [22]. A small number of Oct-4-expressing cells have been observed at the bronchoalveolar junction of the neonatal lung [8]. We have investigated miRNA-mediated modulatory role on Oct-4+ BASCs and have correlated their contribution to SARS infection.

Our analysis establishes a complex interplay between miRNAs, BASCs and certain essential factors related to the disease which might provide a better understanding of SARS pathogenesis. The multifarious relationships shared by genes related to the disease-system pathway, modulatory effects of miRNAs within the prime target cells of infection in response to indefinite cues impose the need of an interaction map. This study develops a complex interaction map between disease-related factors, miRNAs and BASCs in SARS disease pathway, which will provide some clues for diagnostic markers to view an overall interplay leading to disease progression. Further, our investigation towards investigating the therapeutic potential of miRNA-mediated RNAi as an effective antiviral agent against SARS might unravel some of the efficient therapeutic measures against the deadly disease in future.

## Results and Discussion

We have investigated the cellular tropism of SARS-CoV (Table 1) in the BASCs present in lungs and attempted to establish the role of BASC-miRNAs towards understanding the pathogenesis of SARS in lung fibrosis. We describe the role of BASCs as the target cells of SARS infection and show how a repertoire of BASC miRNAs act as molecular switches to contribute to this host-pathogen interaction.

**Table 1:** SARS-CoV strains considered in our study.

Name of the Strains	Accession no.
SARS coronavirus Urbani	AY278741
SARS coronavirus ZJ01	AY286320
SARS Coronavirus CDC#200301157	AY714217
SARS coronavirus Taiwan TC1	AY338174
SARS coronavirus Taiwan TC3	AY348314
SARS coronavirus ZJ0301 from China	DQ182595
SARS coronavirus TOR2	AY274119
SARS coronavirus GD01	AY278489
SARS coronavirus FRA	AY310120
SARS coronavirus Shanhgai LY	AY322207
SARS coronavirus NS-1	AY508724
SARS coronavirus civet007	AY572034
SARS coronavirus civet010	AY572035
SARS coronavirus civet020	AY572038
SARS coronavirus TJF	AY654624
SARS coronavirus B039	AY686864
SARS coronavirus A022	AY686863
SARS coronavirus WH20	AY772062
SARS coronavirus TWH	AP006557
SARS coronavirus TWJ	AP006558
SARS coronavirus TWK	AP006559
SARS coronavirus TWS	AP006560
SARS coronavirus TWY	AP006561
SARS coronavirus Frankfurt 1	AY291315
SARS coronavirus HSR 1	AY323977
SARS coronavirus Sino3-11	AY485278
SARS coronavirus Sino1-11	AY485277
SARS coronavirus ShanghaiQXC2	AY463060
SARS coronavirus ShanghaiQXC1	AY463059
SARS coronavirus LLJ-2004	AY595412

### Primary site of SARS infection

Which is the primary site of SARS infection and how do these corona virus interacts with those cells to enter the host and eventually invades the host immune system to spread itself - is a pertinent question which prevails and is a debated issue. In order to throw light on this very important question, we looked upon at the SARS^+^ cell markers and BASC markers (specific to each of its developmental stages from multipotent state to fully differentiated state).

It has been reported that SARS-infected cells are unlikely to be pneumocytes (both type I or type II) or cytokeratin^+^ epithelial cells and it is quite obvious since SARS-CoV cannot infect or replicate in a fully differentiated cell [23]. Further, SARS^+^ cells are distinct from cells expressing the macrophage/monocyte specific marker CD68. But SARS^+^ cells are found to express the functional receptor ACE2 as well as the stem/progenitor cell marker CD34 and Oct-4 [23]. Chen *et al* have also shown that SARS^+^ cells are CD45^−^. Hence it has been confirmed that the SARS^+^ cells in the infected lung were a subset of putative stem/progenitor cells expressing CD34, Oct-4 and ACE2. Further, it is unlikely for SARS^+^ cells to produce differentiated cell markers. The presence of SARS^+^ cells within the multiple cell types at the bronchiolar lining layers makes it difficult to isolate SARS^+^ cells. Some of the SARS^+^ cells remains overlapped with adjacent cytokeratin^+^ cells which might be mistakenly interpreted as colocalized cells. In such a situation BASC (Sca-1^+^ CD34^+^ CD45^−^ Pecam^−^) in terminal bronchioles located exclusively at the broncho-alveolar duct junction (BADJ) stands a good selection to be investigated as the prime target cells of SARS-CoV infection.

### Characterizing BASC at BADJ as the prime target cell of SARS infection initiation

A percentage of the Sca-1^+^ CD4^−^ Pecam^−^ cell populations at BADJ are CD34^+^. This Sca-1^+^ CD34^+^ CD45^−^ Pecam^−^ population is enriched for BASCs [12] became evident from the fact that it contained no ciliated cells. In addition to clonal colony formation, these cells exhibited extensive self-renewal in culture. They also had a greater capacity for differentiation compared to other lung epithelial cells. Sca-1^+^ CD34^+^ CD45^−^ Pecam^−^ BASC cultures further confirmed the multilineage differentiation capacity of BASCs. Within seven to ten days they show up positive for differentiated cell markers as they get differentiated into Clara-like cells (CCA^+^ SP-C^−^), AT2-like cells (SP-C^+^ CCA) and AT1-like cells (AQ5^+^).

**Figure 1:**
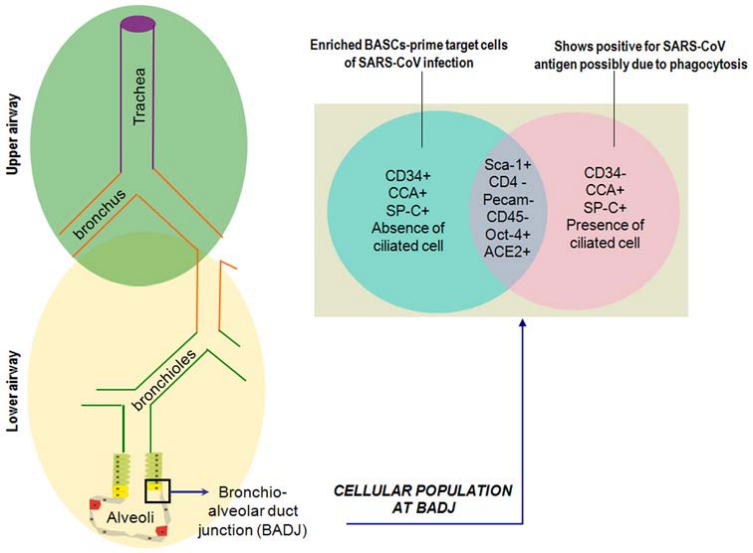
Lower respiratory tract focusing on bronchoalveolar duct junction (BADJ) of the lung. Venn diagram represents an enlarged section of the BADJ showing up the different subsets of the distinct cellular population (Oct-4^+^ ACE2^+^) in this region of the lung. A subset of this cellular population is enriched in BASCs identified as Sca-1^+^ CD45^−^ Pecam^−^ CD34^+^, the other subset being Sca-1^+^ CD45^−^ Pecam^−^ CD34^−^. On differentiation Sca-1^+^ CD45^−^ Pecam^−^ CD34^+^ cells show positive for CCA and SP-C; Sca-1^+^ CD45^−^ Pecam^−^ CD34^−^ cells show positive for CCA, SP-C, and ciliated cells.

Within the Sca-1^+^ CD4^−^ Pecam^−^ cells the other percentage of cells showed CD34^−^ and contained ciliated cells. The Oct-4^+^ colony cells which are Sca-1^+^ CD34^−^ SSEA-1^+^ cytokeratin^+^ were shown to be succeptible to SARS-CoV infection by Ling *et al*. [8]. These might be a subset of Sca-1^+^ CD4^−^ Pecam^−^ CD34^−^ population (Figure 1). Later studies have shown that cytokeratin^+^ cells do not express the functional receptor ACE2 [23]. Further, SARS^+^ cells do not express cytokeratin and are CD34^+^ SSEA-1^−^. Phagocytosis is a common mode of virus entry within surrounding cells in SARS infection [24]. Hence, presence of SARS antigen within the subset of Oct-4^+^ colony cells which are Sca-1^+^ CD34^−^ SSEA-1^+^ cytokeratin^+^ is most likely due to phagocytosis. Thereby, it is evident that these Oct-4 cells (Sca-1^+^ CD34^−^ SSEA-1^+^ cytokeratin^+^) are not the prime target cells for SARS-CoV infection.

On the contrary, the BASCs (Sca-1^+^ CD34^+^ CD45^−^ Pecam^−^) must be another subset of these Oct-4^+^ ACE2^+^ epithelial colony cells (Figure 1) which provides a more favorable environment for SARS-CoV entry and replication. Absence of ciliated cells and CD34^+^ makes them the most probable targets of SARS-CoV at the onset of infection.

### miRNAs as modulators of BASC differentiation

Since SARS-CoV cannot infect or replicate in a fully differentiated cell, it is indeed essential for the virus to control the differentiation stages in an infected BASC cells so that they do not reach a fully differentiated state until the virus has undergone successful transmission and replication within this primary site of infection. Hence, it is important to monitor the expression levels of the different developmental stage specific markers of SARS infected BASC and also that of the corresponding regulatory switches that control their level of expression.

MiRNAs are widespread agents of post-transcriptional gene silencing and have been strongly linked with stem cells [25], [26]. They exhibit a high degree of stage- and tissue-specificity, and therefore it is important to scan the profiling data for those miRNAs that operate during the narrow windows of development of a BASC in order to understand their regulatory impact on the expression levels of essential marker genes and transcription factors within the cell. In order to model the regulatory mechanisms within a SARS^+^ cell, we have tried to understand the underlying regulatory mechanisms within a normal BASC cells mediated by the molecular switches- the miRNAs.

It has been established that the virus exploits the host-miRNA milieu to foster their own agenda of evading the host immune system, thereby establish a consistent infection and continue replication.

### mh–miRNAs targeting host factors

**Figure 2:**
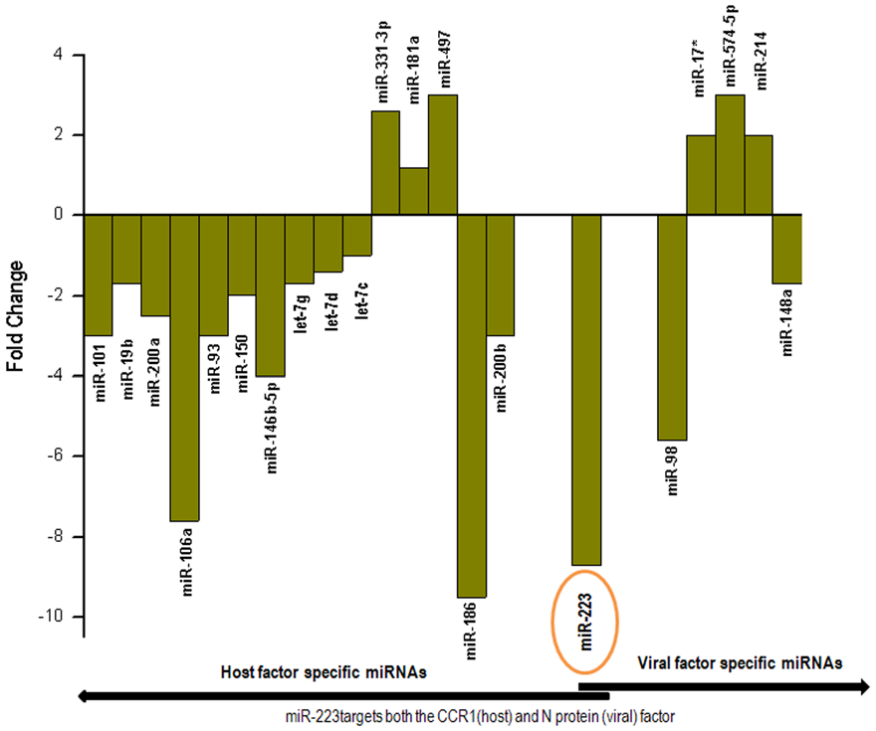
Fold change of the differentially expressed host miRNAs in BASC targeting host and virulent viral factors [61].

We undertook an intensive transcriptome-wide search for candidate mh-miRNAs (BASC-miRNAs which are homologous between mouse-human whole genomes) (Table 2) targeting the developmental stage specific transcriptional factors and marker genes of BASCs. Table 3 shows the list of the miRNAs targeting the potential stage specific factors of BASCs. Further, we have also elucidated the mh-miRNAs targeting ACE2. The target factors have further been mapped with the expression levels of their corresponding miRNAs within BASCs which shows their integrated correlation contributing towards each developmental stage of BASCs in a healthy individual (Figure 2). SARS-CoV definitely interferes with these miRNA mediated regulation to dictate the cell fate and continue its replication. Hence as a future advancement of this work, it will be worthy designing benchwork to detect the correlation of these miRNA-mRNA expressions within a SARS-CoV infected cell.

**Table 2:** Significantly expressed mh–miRNAs in bronchio-alveolar stem cells.

miRNAs	Mouse Accession No.	Human Accession No.
miR-142-3p	MIMAT0000155	MIMAT0000434
miR-19a	MIMAT0000651	MIMAT0000073
miR-144	MIMAT0000156	MIMAT0000436
miR-374	MIMAT0003727	MIMAT0004955
miR-7a	MIMAT0000677	MIMAT0000252
miR-186	MIMAT0000215	MIMAT0000456
miR-15b*	MIMAT0004521	MIMAT0004586
miR-340-5p	MIMAT0004651	MIMAT0004692
miR-223	MIMAT0000665	MIMAT0000280
miR-451	MIMAT0001632	MIMAT0001631
miR-106a	MIMAT0000385	MIMAT0000103
miR-140	MIMAT0000151	MIMAT0000431
miR-421	MIMAT0004869	MIMAT0003339
miR-142-5p	MIMAT0000154	MIMAT0000433
miR-20b	MIMAT0003187	MIMAT0001413
miR-98	MIMAT0000545	MIMAT0000096
miR-92a	MIMAT0000539	MIMAT0000092
miR-15b	MIMAT0000124	MIMAT0000417
miR-146b	MIMAT0003475	MIMAT0002809
miR-20a	MIMAT0000529	MIMAT0000075
miR-301a	MIMAT0000379	MIMAT0000688
miR-148b	MIMAT0000580	MIMAT0000759
miR-15a	MIMAT0000526	MIMAT0000068
miR-218	MIMAT0000663	MIMAT0000275
miR-17	MIMAT0000649	MIMAT0000070
miR-106b	MIMAT0000386	MIMAT0000680
miR-25	MIMAT0000652	MIMAT0000081
miR-486	MIMAT0003130	MIMAT0002177
miR-193	MIMAT0000223	MIMAT0000459
miR-151-5p	MIMAT0004536	MIMAT0004697
miR-93	MIMAT0000540	MIMAT0000093
miR-101b	MIMAT0000616	MIMAT0000099
miR-127	MIMAT0000139	MIMAT0000446
miR-483	MIMAT0004782	MIMAT0004761
miR-574-5p	MIMAT0004893	MIMAT0004795
miR-21	MIMAT0000530	MIMAT0000076
miR-200b	MIMAT0000233	MIMAT0000318
miR-342-3p	MIMAT0000590	MIMAT0000753
miR-151-3p	MIMAT0000161	MIMAT0000757
miR-429	MIMAT0001537	MIMAT0001536
miR-335-5p	MIMAT0000766	MIMAT0000765
miR-16	MIMAT0000527	MIMAT0000069
miR-181d	MIMAT0004324	MIMAT0002821
miR-99b	MIMAT0000132	MIMAT0000689
miR-146a	MIMAT0000158	MIMAT0000449
miR-331-3p	MIMAT0000571	MIMAT0000760
miR-574-3p	MIMAT0004894	MIMAT0003239
miR-10a	MIMAT0000648	MIMAT0000253
miR-200a	MIMAT0000519	MIMAT0000682
miR-423-5p	MIMAT0004825	MIMAT0004748
miR-17*	MIMAT0000650	MIMAT0000071
miR-671-5p	MIMAT0003731	MIMAT0003880
miR-126-3p	MIMAT0000138	MIMAT0000445
miR-125a-5p	MIMAT0000135	MIMAT0000443
miR-92b	MIMAT0004899	MIMAT0003218
let-7d*	MIMAT0000384	MIMAT0004484
miR-27a	MIMAT0000537	MIMAT0000084
miR-214	MIMAT0000661	MIMAT0000271
miR-150	MIMAT0000160	MIMAT0000451
miR-652	MIMAT0003711	MIMAT0003322
miR-210	MIMAT0000658	MIMAT0000267
miR-18a	MIMAT0000528	MIMAT0000072
miR-361	MIMAT0000704	MIMAT0000703
miR-152	MIMAT0000162	MIMAT0000438
miR-24	MIMAT0000219	MIMAT0000080
miR-148a	MIMAT0000516	MIMAT0000243
let-7g	MIMAT0000121	MIMAT0000414
miR-19b	MIMAT0000513	MIMAT0000074
miR-130b	MIMAT0000387	MIMAT0000691
miR-128	MIMAT0000140	MIMAT0000424
miR-181b	MIMAT0000673	MIMAT0000257
miR-29b	MIMAT0000127	MIMAT0000100
miR-455	MIMAT0003742	MIMAT0004784
miR-221	MIMAT0000669	MIMAT0000278
miR-30c	MIMAT0000514	MIMAT0000244
miR-27b	MIMAT0000126	MIMAT0000419
miR-30a	MIMAT0000128	MIMAT0000087
miR-320	MIMAT0000666	MIMAT0000510
miR-23b	MIMAT0000125	MIMAT0000418
let-7d	MIMAT0000383	MIMAT0000065
miR-23a	MIMAT0000532	MIMAT0000078
miR-125b-5p	MIMAT0000136	MIMAT0000423
miR-30d	MIMAT0000515	MIMAT0000245
let-7a	MIMAT0000521	MIMAT0000062
miR-199a-5p	MIMAT0000229	MIMAT0000231
miR-26a	MIMAT0000533	MIMAT0000082
miR-191	MIMAT0000221	MIMAT0000440
miR-30e	MIMAT0000248	MIMAT0000692
miR-30b	MIMAT0000130	MIMAT0000420
miR-26b	MIMAT0000534	MIMAT0000083
miR-181a	MIMAT0000210	MIMAT0000256
miR-143	MIMAT0000247	MIMAT0000435
miR-195	MIMAT0000225	MIMAT0000461
miR-145	MIMAT0000157	MIMAT0000437
let-7c	MIMAT0000523	MIMAT0000064

mh-miRNAs- mouse miRNAs having complete homology with human miRNAs.

**Table 3:** Predicted miRNA–mRNA pairs within the host system.

Target Genes	mh-miRNAs	Functions
**Class A**		
SCA1 (ATXN1)	miR-101miR-19bmiR-200amiR-106amiR-93miR-150	Stem Cell Antigen-1 is identified as a potential marker for BASCs located at the BADJ [12], [27].
OCT4 (POU5F1)	miR-146b-5p	Octamer-binding transcription factor 4+ (Oct-4) is a stem cell marker protein. It acts as a master switch in differentiation by regulating cells that have pluripotent potential. Recently, it has been found to be expressed in pulmonary cells which are a target for SARS infection [8], [23].
CD34	let-7glet-7dlet-7c	These are membrane-bound stem cell markers for BASCs. This is also expressed in SARS-infected lung cells [23], [28].
**Class B**		
SP-C (SFTPC)	miR-331-3p	Marker protein of alveolar type-2(AT2-like) cells which is a subset of the differentiated BASC population. Hence is quiescent in normal lung but proliferate in response to bronchiolar and alveolar epithelial injury [29], [30].
CCSP-2	miR-181amiR-497	This is the Clara cell marker protein. These are also quiescent in normal lung and proliferate in response to bronchiolar and alveolar epithelial injury [30].
Aquaporin5	miR-331-3pmiR-497	Marker protein of AT1-like cells which is another subset of the differentiated BASC population. Aquaporin5 plays a critical role in the maintenance of normal lung water homeostasis [30], [31].
**Class C**		
ACE2	miR-186miR-93miR-200b	Angiotensin-converting enzyme 2 (ACE2), the primary receptor of SARS-CoV on the host cell. It is a negative regulator of the rennin-angiotensin system (RAS) in the setting of acute lung injury and in response to pulmonary infection with the SARS-CoV [17], [32].
CCR1	miR-223	Inflammatory chemokine receptor for CCL3 and CCL5 & responsible for lung fibrosis [33], [34].

We have predicted the differentially expressed mh–miRNAs which operate within the narrow windows of development of BASCs by targeting a set of host encoded mRNAs.

**Class A** specifies the host mRNAs corresponding to BASC marker proteins which designates its undifferentiated state as well as the state of proliferation and/initiation of differentiation.

**Class B** specifies the host mRNAs corresponding to BASC marker proteins as it reaches a fully differentiated state.

**Class C** specifies the host mRNAs corresponding to the receptor proteins which participates in SARS-CoV pathogenesis.

These miRNA–mRNA pairs specific to BASC are important to understand host-virus interaction network.

### Host miRNA targets in viral genome

We have searched for the host miRNA targets within the virulent genes of SARS-CoV. Table 4 includes the BASC-miRNA candidates targeting the most important virulent proteins responsible for viral infection. Interestingly, miRNAs-17*, -574-5p and -214 targets all the four viral virulent proteins viz. S, N, M, E and orf1a. miR-148a has its target in ORF1a, E, S and M. miR-223 and miR-98 has an exclusive correlation with their targets within the 3′ UTRs of N and S respectively (Figure 2).

**Table 4:** Host miRNAs targeting virulent protein coding mRNAs of SARS-CoV.

Target genes	mh-miRNAs	Functions of target genes
Spike (S)	miR-214miR-574-5pmiR-17*miR-148amiR-98	Responsible for viral attachment and entry into host cells by interacting with ACE2 [5], [35]
Envelop (E)	miR-574-5pmiR-214miR-17*miR-148a	E is responsible for virion envelope morphogenesis and acts as a viroporin, inducing the formation of hydrophilic pores in cellular membranes [36]
Membrane (M)	miR-574-5pmiR-214miR-17*miR-148a	Induction of apoptosis [37]
Nucleocapsid (N)	miR-574-5pmiR-214miR-17*miR-223	Promotes tissue fibrosis [19]N protein inhibit cell cycle progression [38], [39]
ORF1a	miR-574-5pmiR-214miR-17*miR-148a	Encodes viral replicase proteins [40], [41]

Normally presence of such host miRNA targets in SARS-CoV genome indicates that the corresponding miRNAs are a part of the host's innate antiviral defense. On the contrary, the RNA virus co-opt host-miRNAs to suppress their own replication to evade immune elimination and establish a strong infection. It has been found that human infecting single-stranded RNA viruses are enriched for target sequences of human miRNAs [42]. Or else rapidly evolving RNA viruses might evolve targets that match host miRNAs in order to increase their host specificity. It is very likely that the rapidly evolving virus take advantage of slowly evolving host miRNAs to increase their survival in the host, analogous to the manner in which viruses take advantage of other cellular processes. Co-option of host miRNAs by SARS-CoV to suppress their own replication will allow it to be in a latent state and escape the host immune system at the initial time point of transmission and infection. Once successful transmission within the host system takes place, antiviral miRNA target sequences in the virus can be expected to mutate rapidly to maximize mismatches and thus minimize the impact of ‘antiviral’ miRNAs. Rapid mutation in SARS-CoV is supported by the fact that SARS-CoV lack proof reading activity in their polymerases. RNA-dependent polymerase in SARS-CoV misincorporates 8.26 bases per million [43].

**Figure 3:**
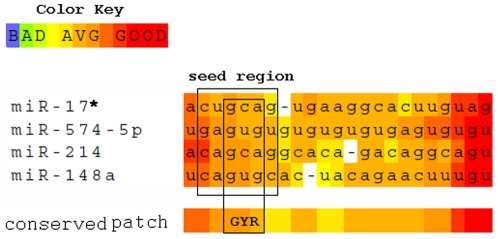
Sequence alignment between the common viral specific host miRNA mature sequences from 5′ to 3′ end. The seed region (position 2 to 7) and the conserved positions (3^rd^, 4^th^, and 5^th^) within the seed region are highlighted within boxes.

While investigating the seed sequence conservation of the viral-specific host miRNAs (Figure 3) we found that there is a strong bias towards G, Y(pYrimidine) and R (PuRine) bases at the 3^rd^, 4^th^ and 5^th^ position respectively of the seed region. This implies that a single mutation within the complementary regions of this conserved 3-mer motif within the viral targets will disrupt the miRNA seed-target match and will enable the virus to escape the antiviral effect of host-miRNAs after successful transmission within the host. Such host-miRNA sequence conservation helps the virus to mutate the target regions with more precision and at ease so as to escape the effect of all the 4 miRNAs together.

The miRNA expression profiles of three miRNAs-17*, -574-5p and -214 targeting S, N, Orf1a, M and E shows that they are upregulated within uninfected BASCs. Presumably at the onset of SARS infection the expression of these targets is suppressed which is likely to control viral replication and consequently help the virus to evade the host immune system. But the targets for downregulated miR-148a within ORF1a, M, S and E might escape repression and maintain replication at a lower rate. Further, the exclusive miRNAs viz. miR-223 and -98 for N and S protein respectively are highly downregulated (8.7 fold and 5.6 fold respectively) in BASCs. N and S proteins are important for viral transmission in host. This provides a clue towards the fact that the S protein and N protein (having targets corresponding to these miRNAs) of SARS-CoV takes advantage of this and escapes miRNA-mediated repression which is a rescue to the virus for effective transmission at the initial stage of viral infection.

### Key miRNA–mRNA pairs correlating with virus entry, replication, and host–virus interaction

#### ACE2-S interaction

It has been shown that proinflammatory and fibrogenic cytokine pathways are activated within the first 24–48 hr following pulmonary insult resulting from SARS-CoV infection [44]. High initial levels of these activities are associated with persistent pulmonary damage and increased risks of subsequent pulmonary fibrosis and poor outcome in diffuse alveolar damage. Further, it has been reported that ACE2 mRNA, protein and enzymatic activity is severely decreased in pulmonary fibrosis resulting from SARS-CoV infection [17], [18]. Studies have also been performed which support that differential regulation of host cell mRNA transcription and protein synthesis occurs in a SARS-CoV infected cells [45].

SARS-CoV S protein is a multifunctional protein which plays pivotal roles in the biology and pathogenesis of SARS-CoV. It has been shown that S protein mediates viral infection by binding to cellular receptor ACE2 and thus induces membrane fusion. SARS-CoV infections and the Spike protein of the SARS-CoV reduce ACE2 expression [46]. Further a recent work of Imai *et al*. showed that on injecting SARS-CoV Spike into mice worsens acute lung failure in vivo, which can be attenuated by blocking the renin–angiotensin pathway. This suggests that the S-protein mediated activation of the pulmonary RAS influences the pathogenesis of ALI/ARDS and SARS [47]. But the molecular mechanism by which S protein down regulates ACE2 in lung cells following SARS injury is not well addressed till to date.

**Figure 4:**
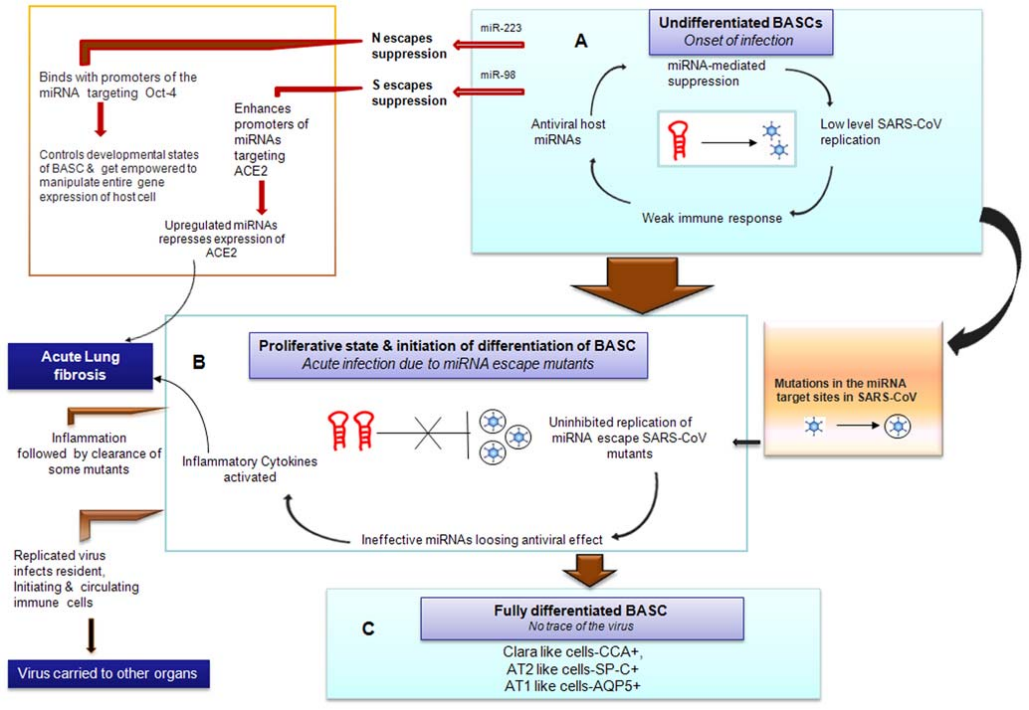
Modelled miRNA–mediated host–virus interaction within BASC in SARS-CoV pathogenesis. Host miRNAs are central to viral pathogenesis. This figure illustrates how the host miRNA and SARS-CoV interactions can explain the features of SARS-CoV pathogenesis such as (A) entry of the virus in an undifferentiated BASC, (B) causing acute infection during the proliferative stage and onset of BASC differentiation, and (C) complete clearance of virus in a fully differentiated BASC.

In our work we observed that the miRNAs- miR-186,-93 and -200b targeting ACE2 are found to be highly down regulated in normal BASCs thereby showing that they do not interfere with the expression level of ACE2 and thus prevents lung fibrogenesis by limiting the local accumulation of the profibrotic peptide ANG II in an uninfected state. Considering the expression of S-protein, we find that its target corresponding to highly downregulated miR-98 probably escapes miRNA-mediated host RNAi defense at the onset of infection and interferes with ACE2 expression. Figure 4 shows how the virus takes advantage of the microRNA-mediated complex interplay between the host and viral factors within BASCs. The question still remains regarding the molecular mechanism by which this S-protein represses the expression of ACE2.

S-proteins have been shown to act as transcription factor activating the promoters of GRP94/78 genes inducing ER stress in a SARS-CoV infected cell [48]. The role of S protein as a transcription factor has obliged us to think that they might play a role to activate the miRNA promoters of miR-186,-93 and -200b targeting ACE2 to enhance their expression resulting in a reduced expression of ACE2 in an infected host. Whether this decrease in ACE2 expression is a result of miRNA mediated gene silencing controlled by S-protein will be an interesting and compelling question for further study.

#### Oct-4-N interaction

Oct-4 plays a crucial role towards regulating the chromatin structure in a state consistent with self-renewal and later facilitating the expression of genes that keeps the cell poised to respond to cues that lead to differentiation. Hence this is one of the essential factors which control the state of differentiation in BASCs. Furthermore, it has a significant role in apoptosis. miR-146b-5p is seen to target Oct-4 in normal BASCs. The downregulated expression of this miRNA explains for the uninterrupted expression of Oct-4 resulting its normal function in an uninfected cell. We have harnessed the high possibility of a complicated crosstalk between the ability of N-protein to control the expression of Oct-4 in an infected cell.

N protein is known to be the most abundantly expressed protein of SARS-CoV. The most unique and significant property of it is revealed in its ability to act as a sequence specific DNA binding factor. It has been reported to bind NFkB response element of COX2 promoter and enhance COX2 gene expression [49] which effectively is beneficial for its existence within the host. This reveals the power of the N-protein to manipulate the entire gene expression programme of the infected cell. The common miRNAs, miR-214,-574-5p and -17* having targets in N protein are upregulated in BASCs. But miR-223, the unique miRNA targeting N-protein is highly downregulated. This signifies that the N-protein targets corresponding to miR-223 is going to escape miRNA-mediated downregulation at the onset of infection (See Figure 4). Further it seems that N-protein plays a role in interfering with the expression modulation of Oct-4 by binding to the promoter of miR-146-5p and controls the different stages of BASC differentiation as well as prevents apoptosis of SARS infected cells.

### Immunity-related issues—induction of inflammatory chemokines in SARS-CoV–infected cells might be an advantage for the virus to replicate

All respiratory virus disease results from two concurrent pathological components: ongoing virus replication and the resulting inflammatory response. Even if antivirals clearly inhibit virus replication, the biochemical and cellular inflammatory responses to the initial infection related events continue despite diminished virus titer [50]. While acute inflammatory responses are generally beneficial in nature and have been shown to limit virus replication in situ, prolonged, uncontrolled inflammation has been recognized as a significant component contributing to the pathological sequelae to the morbidity and mortality of severe acute respiratory syndrome coronavirus infection [44]. A histopathologic analysis of SARS-CoV infected lung in mice showed that there has been an induction of significant changes in the expression levels of the chemokine receptor CCR1 which acts as receptors for CCL3 and CCL5. The expression levels of these inflammatory chemokines were increased by viral infection of the lung [33]. It has been shown that CCR1 play an essential role in the pathogenesis of pulmonary fibrosis in lung-injury models [34]. Further, previous work suggests that antagonism of signaling via the chemokine receptor CCR1 is a potent strategy that attenuates leukocyte recruitment in response to severe respiratory virus infection [50].

Although there has been a subsequent increase in inflammatory cytokines related to innate immune response in response to viral infection, there has been a lack of immunoregulatory cytokines (T or B lymphocytes) in the lung during infection which confirms the fact that the expression of viral replication proteins remains at a quiescent state so as to avoid viral clearance by the activation of immunoregulatory cytokines by adaptive immune responses at the onset of the infection. On the contrary, activation of inflammatory chemokines might be acting as a beneficial phenomenon for the replication of various viruses [51], [52].

Emerging studies suggest that miRNAs provide an added layer in orchestrating immune responses [53]. miRNAs function in shaping immunity by regulating the repertoire of genes expressed in immune cells and the magnitude and duration of responses to particular pathogens [54]. Hence we investigated the underlying miRNA induced molecular mechanisms responsible for these changes in the lung and we found that miR-223 has its target in the 3′-UTR of CCR1. Previously, miR-223 was experimentally identified in chorioamnionitis-related inflammation and its predicted target genes include several genes involved in inflammation and immune responses. Further, miR-223 is known to promote granulocyte differentiation and is a part of a regulatory loop that involves C/EBP and NFI-A [55].

Interestingly miR-223 has its target in N protein of SARS-CoV too and it is one of the miRNAs which probably helps the virus to escape the RNAi mediated silencing at the onset of infection. Or in another way we may say that the virus encodes the target of miR-223 to escape the RNAi mediated repression and at the same time takes advantage of the effect of induction of the inflammatory chemokine responses to accelerate lung fibrosis. But the controlled expression of target CCR1 in an uninfected cell inspite of the significantly low expression of its corresponding miR-223 in BASCs suggest that there must be a cascaded fine tuned interaction masterminded by the virus which is going on within the infected cell. It is known that N protein activates NF-KB in Vero E6 cells [38]. CCR1 is one of the target genes of NF-KB which is activated at a later stage [56]. Hence, we propose that within BASCs, reduced expression of miR-223 helps N protein to enter the host cell. On successful entry, N protein might be using miR-223 once again to activate CCR1 via NF-KB at its replicative stage to enhance lung fibrosis and at the same time gets empowered to manipulate the entire gene expression of the host cell by controlling the expression of Oct-4. The exact mechanism of such miRNA-mediated events needs further investigation. Further, checking the expression levels of N-protein, CCR1 as well as miR-223 in an infected BASC will provide a more compact conclusion.

### Conclusion

Based on these findings, we propose that BASCs (Sca-1^+^ CD34^+^ CD45^−^ Pecam^-^) are a subset of Oct-4^+^ ACE2^+^ cells and are the chief targets of SARS-CoV infection. SARS-CoV enters the host; infects the BASCs of the lungs and controls its developmental stages via the molecular switches-miRNAs. Co-option of host miRNAs by viruses reveal their intelligent plan to control their replication in order to evade immune elimination until they undergoes successful transmission and establish a strong infection. Thereafter, they undergo rapid mutation to maximize the target-miRNA mismatches and enhance their replication before the cell reaches at a fully differentiated state. On successful replication, the virus infects resident, infiltrating, and circulating immune cells. The circulating immune cells carry the virus to other organs and causes damages to the immune cells of spleen, peripheral and central lymph nodes and other lymphoid tissues. The immune defense being weakened significantly, leads to rapid deterioration of the pneumonia. It is worth to test these cells for the expression of Oct-4 and ACE2 for further confirmatory conclusion.

The proposed host miRNA-dependent mechanism probably acts in concert with the host and viral factors for establishing a strong infection. Our proposal shifts away from the simplistic notion that host miRNAs with specific sequence complementarity to viruses are indicative of a bonafide innate antiviral immune mechanism to an established viral pathogen.

Although the immediate threat of SARS is over, we intend to continue with its therapeutic measures partly to guard for its possible return. Moreover, this will help us to transform our knowledge from SARS to other emerging viral diseases specially causing lung injuries which most certainly will be a deadly threat in future. Lot of attempts have been made and is still going on to design effective antiviral agents against this deadly disease [57]–[60]. While some of these approaches showed partial efficiency to combat SARS infection, the others need further clinical investigations to be used as a proper therapeutic reagent.

This era has witnessed an increase in the development of exogenous siRNA therapeutics against viruses. The potential therapeutic effect of modulating host-miRNA levels is also worth considering. This emerging picture of miRNA regulation in SARS-CoV infected individual will make the host-virus interaction far richer and more complex than the crisp linear pathways of the previous decade, with miRNAs participating in executive decisions at the interesting and vulnerable nodes in regulatory networks. By boosting the level of the host virus-specific miRNAs, it might be possible to turn what was a desirable outcome for the virus into a desirable outcome for the patient. Under the current hypothesis, we would predict that decreasing the level of host virus-specific miRNAs would result in promotion of viral replication, leading to immunological recognition and clearance of the virus by the host immune system. Further, increasing the level of miRNAs exclusive for the N and S protein might also block viral entry. However, manipulating the level of host miRNAs could have unintended consequences because the physiological functions of the miRNAs might be altered or viral pathology might be enhanced. Further silencing of miRNAs with antagomirs specifically within the infected cell could become another therapeutic strategy where miRNAs participate in disease aetiology. Nevertheless, these potential interventions merit further evaluation.

## Materials and Methods

To identify the miRNAs involved in the cellular tropism of SARS-CoV in lungs, we undertook an intensive search for potential miRNA candidates in the BASCGAP (Broncho-alveolar stem cell genome anatomy project) sequence data. Our miRNA sequences were derived from the Sanger Institute miRBase release 12.0 (http://microrna.sanger.ac.uk/sequences). The global miRNA expression profile of our BASCGAP source sequences were derived from mouse BASCs [61].

### miRNA—mouse-human (mh–miRNA)

Our search strategy was motivated by the need to apply pure-homology based selection of miRNAs so that the results represent a closer approach to mimic the molecular mechanisms that underlie disease pathway behaviors and response in a human host. Further the overall miRNA expression profile of mouse and human lungs are similar [62]. From the BASCGAP sequence data of 116 miRNAs, we screened out the BASC-miRNAs lying at the intersection of mouse-human whole genomes. This filter is based on a homology search (SSEARCH), performed with miRBase (release 12.0) and is applied on the mature candidates from the BASCGAP sequence data. E-value cutoff considered for SSEARCH was <10.0. Mature miRNA sequence homology was one of the stringent parameters considered in our search. Table 2 represents the screened output set of 95 mature candidates termed as mh-miRNAs.

### mRNA targets

We have considered the developmental stage specific marker proteins of BASCs, which includes the transcription factor Pou5f1; ACE2-the most important receptor of SARS-CoV (Table 3). In addition to these, we have chosen the virulent protein coding genes, viz. Nucleocapsid (N), Spike(S), Envelope (E), Membrane (M) and Orf1a from 30 strains of SARS-CoV (Table 1 and Table 4) for our investigation. Annotated UTR sequences of the target genes were retrieved from UTRdb [63].

### Target prediction

We adopted RNAhybrid [64], that predicts the most favorable hybridization sites between miRNA and UTR regions and generates energy minimized duplex structures using the Dynamic Programming technique. The resultant miRNA-mRNA duplexes are consistent with the following structural and energy constraints at the first step of screening the targets: (i) Binding energy cutoff < = −20 kcal/mol [64]; (ii) complementarity of minimum seven bases to the 5′ end of the miRNA; (iii) minimization of GU base pair within the miRNA-target duplex, a maximum of only one GU base pair between 2 and 7 position of the miRNA is allowed; (iv) minimization of loop size throughout the duplex.

Although, currently available target prediction methods are diverse, both in approach and performance and all have room for further improvement, perfect seed pairing centered on nucleotides 2–7 is considered as the most important criteria for prediction reliability. Furthermore, in order to screen out the most favorable target in the case of miRNAs having multiple targets we incorporated the constraint of site efficacy (hierarchy of site efficacy follows- 8 mer>7 mer-m8>7 mer-A1>6 mer) following the work of Grimson *et al.* and Friedman *et al*. [65], [66]. 3′ compensatory pairing of miRNA to its target have been considered to compensate for imperfect/weak seed matches [67]. We have further checked the effect of cooperativity as well as the UTR binding location.

### miRNA–mRNA enrichment analysis

For *bantam* miRNAs, good minimum free energies (MFEs) can occur frequently by chance. The longer a putative target sequence, the better such random energies will be. Hence, statistical significance of predicted targets is assessed with an extreme value statistics of length normalized minimum free energies and a Poisson approximation of multiple binding sites.

### Microarray data analysis in BASCs

**Figure 5:**
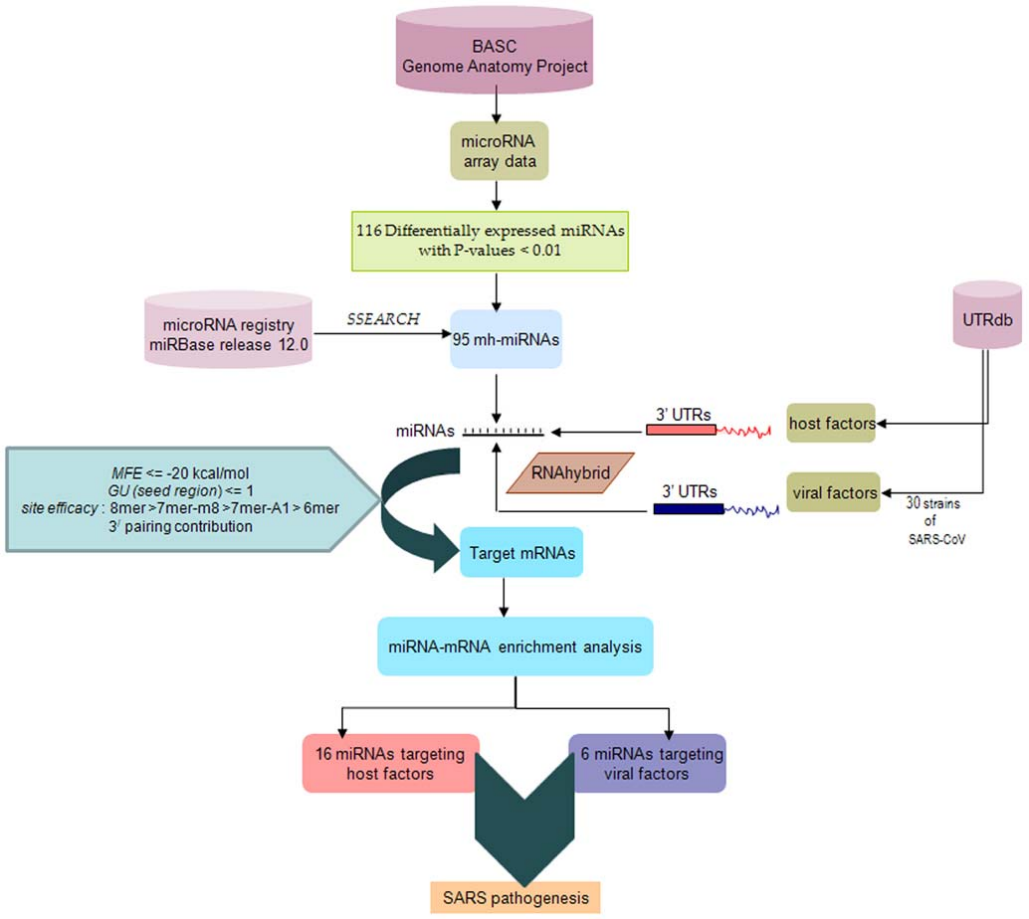
miRNA–mRNA prediction pipelines. This flow chart summarizes the steps followed and yields from BASCGAP consortium sequences to predict the miRNA-mRNA pairs involved in SARS pathogenesis.

miRNA expression profile of 2×10^6^ mouse BASCs (CD45^−^CD31^−^CD34^+^ Sca-1^+^) and 4×10^6^ control cells(CD45^−^CD31^−^CD34^−^Sca-1^−^) are determined using miRNA microarray. The data considered for our analysis is obtained by first subtracting the background and then normalizing the signals using a LOWESS filter (locally weighted regression). Background is determined using a regression-based background mapping method [68]. The miRNA dataset was filtered according to the standard procedure to exclude spots with minimum intensity and size. Further for the dual-sample experiments, the ratio of the two sets of detected signals (log2 transformed, balanced) are calculated. Thereafter, t-values are calculated for each miRNA between the test and the control groups, and P-values are computed from the theoretical t-distribution. miRNAs with P-values below a critical P-value (typically 0.01) are the differentially detected signals and were selected as our global miRNA expression profile data set (of mouse BASCs) in BASCGAP. The flowchart of methodology is provided in Figure 5.
